# Supplementary material for: First Molecular Detection and Epidemiological Analysis of Equine Influenza Virus in Two Regions of Colombia, 2020–2023
Source: Viruses. 2024 May 24;16(6):839. doi: 10.3390/v16060839 (PMC11209042; doi:10.3390/v16060839)
Supplement: Supplementary file 1 [file viruses-16-00839-s001.zip › viruses-2974530-supplementary.pdf]

Supplementary Materials

First molecular detection and epidemiological analysis of equine influenza virus in two regions of Colombia, 2020-2023.

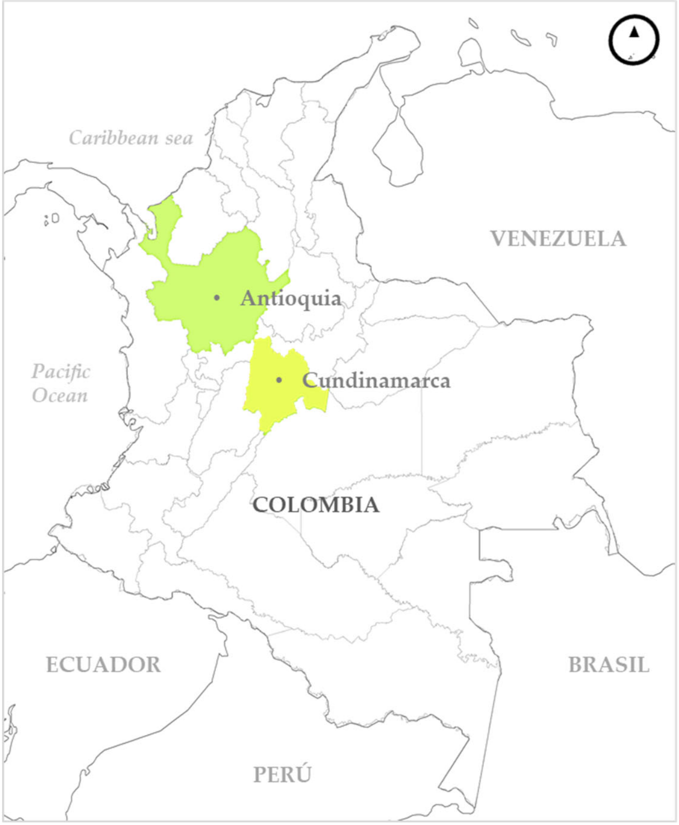

Supplementary Figure S1. Geographical location of study area in Colombia. Map showing the departments of Antioquia and Cundinamarca.

Supplementary Table S1. Descriptive analysis

Categories	Percentage /proportion
1. Activity	
Competition	16% (30/188)
Horse breed	28% (53/188)
Horseback riding	40% (76/188)
Work	4.8% (9/188)
equine therapy	3.2 % (6/188)
Abattoir	1.6% (3/188)
Foal	5,9% (11/188)
2. Vaccination state	
Without Vaccine	85.6 (161/188)
Vaccinated	12.8 (24/188)
Unknown vaccination	1.6(3/188)
3. Breed	
Colombian Creole horse	82.4 % (155/188)
Argentine Polo	4.8% (9/188)
Pony	3.7% (7/188)
Pure Spanish horse	3.2% (6/188)
Friesian	2.7% (5/188)
Arabian	1.1% (2/188)
Mule	1.1% (2/188)
Lusitano	0,5% (1/188)
Gypsy	0,5% (1/188)
4. Sex	
Female	71,8% (135/188)
Male	28,2% (53/188)
5. Age	
5<	68,6% (129/188)
5>	31,4% (59/188)
Clinical signs	
6. Fever	
No	74,5% (140/188)
Yes	25,5 % (48/188)
7. Weight loss	
No	82,4 % (155/188)
Yes	17,6% (33/188)
8. Respiratory distress	
No	91,5% (172/188)

Yes	8,5% (16/188)
9. Presence of Nasal Secretion	
Yes	96,3% (180/188)
No	3,7% (8/188)
10. Dry cough	
Yes	77,7% (146/188)
No	22,3% (42/188)
11. Productive cough	
Yes	6,9% (13/188)
No	93.1% (175/188)
12. Decrease in food consumption	
Yes	29.8 % (56/188)
No	70,2% (132/188)
13. Increase in the size of retropharyngeal nodes	
No	69.7% (131/188)
Yes	30.3% (57/188)
14. Eye discharge	
No	94.7% (178/188)
Yes	5.3 % (10/188)
15. Decreased performance	
No	71,8% (135/188)
Yes	22,3% (42/188)
Has not started treatment	5,9% (11/188)
16. Treatment	
Mucolytic	56,9% (107/188)
Without treatment	29,8% (56/188)
Antibiotic and NSAIDs	9,0% (17/188)
NSAIDs	4,3% (8/188)
17. Location	
Antioquia	64,4% (121/188)
Cundinamarca	35,6% (67/188)
18. Live with dogs	
Yes	96,8% (182/188)
No	3,2% (6/188)
19. Live with cats	
Yes	46,8% (88/188)
No	53,2% (100/188)
20. Live with birds	
Yes	28,7% (54/188)
No	71,3% (134/188)
21. Live with pigs	

Yes	12,2% (23/188)
No	87,8% (165/188)
22. Predominant breed on the farm	
Colombian Creole horse	96,3% (181/188)
Pure Spanish horse	3,2% (6/188)
Lusitano	0,5% (1/188)
23. Equine Density	
1-60	67% (126/188)
61-120	19% (38/188)
121-<	13% (24/188)
24. start of vaccination on the farm	
No vaccine/does not apply	85,6% (161/188)
12 months	7,4% (14/188)
6 months	4,3% (8/188)
Just for move	1,1% (2/188)
unknown vaccination	1,6% (3/188)
25. type of dewormer	
Anthelmintics	50,5% (95/188)
They alternate ivermectin and anthelmintics	24,5% (46/188)
Ivermectin	23,4% (44/188)
unknow	1,6% (3/188)
26. Frequency of dewormer	
3 months	75,5% (142/188)
6 months	14,9% (28/188)
4 months	3,2% (6/188)
Owner decides according to recommendation	6,4% (12/188)
27. Type of disinfectant applied to surfaces	
Hypochlorite	27,1% (51/188)
Creolin	22,3% (42/188)
Do not disinfect	13,8% (26/188)
Detergent	12,8% (24/188)
Ammonium 10%	11,7% (22/188)
glutaraldehyde	9.0% (17/188)
Cal	3,2% (6/188)
28. Pregnant mare vaccination	
Yes	26,1% (49/188)
No	73% (139/188)
29. Frequency of Veterinary Medical Visit	
If a problem arose	54,8% (103/188)
Permanent	45,2% (85/188)

30. lives on property that attends competitions	
Yes	38,3% (72/188)
No	61,7% (116/188)
31. lives on property that attends horseback riding	
Yes	15,4% (29/188)
No	84,6% (159/188)
32. lives on property that attends horseback riding and competition	
Yes	21,3% (40/188)
No	78,7% (148/188)
33. Entry of new horses	
Yes	96.8% (182/188)
No	3.2% (6/188)
34. Use of medical records	
Yes	68,6% (129/188)
No	31,4% (59/188)
35. Separates symptomatic horses	
Yes	11,2% (21/188)
No	88,8% (167/188)
36. Quarantine	
Yes	10,6% (20/188)
No	89,4% (168/188)
37. Molecular diagnostic	
Positive	33,5% (63/188)
Negative	66,5% 125/188)

Supplementary Table S2: Complete Bivariate analysis

Variable	OR Crude	I.C		P value
Competition activity	2,7	1,22	5,99	0,12
Influenza vaccination	0,7	0,60	1,28	0,36
Breed	2,1	0,86	5,15	0,10
Sex	1,6	0,78	6,51	0,20
Fever presence	3,2	1,66	6,51	0,01
Weight loss	1,6	0,74	2,43	0,23
Respiratory distress	0,89	0,29	2,69	0,84
Decrease in food consumption	1,80	0,93	3,42	0,77
increased lymph node size	4,6	2,30	9,01	0,00
Eye discharge	0,48	0,10	2,32	0,35
Decreased performance	1,78	0,91	3,45	0,09
Location	0,77	0,41	1,47	0,42
Share space with Dogs	1,00	0,18	5,69	0,67
Share space with Cats	1,54	0,83	2,85	0,16
share space with birds	2,4	1,26	4,68	0,007
Share space with pigs	0,85	0,33	2,91	0,73
Equine density	0,75	0,14	1,52	0,48
Start of vaccination in the farm	0,28	0,06	1,23	0,76
Pregnant mare vaccination	0,64	0,31	1,32	0,22
type of disinfectant on surfaces	0,72	0,36	1,42	0,35
Veterinary Medical Visit	1,90	1,01	3,54	0,044
lives on property that attends competitions	1,62	0,87	3,17	0,21
lives on property that attends saddle type	0,36	0,13	1,00	0,44
lives on property that attends saddle type and competition	2,57	1,25	5,28	0,01
Entry of new horses	1,82	0,88	3,77	0,10
Separates symptomatic horses	11,9	1,54	90,16	0,003
Quarantine	8,5	1,09	65,54	0,010
